# Behavior change following a self-management intervention for housebound older adults with arthritis: an experimental study

**DOI:** 10.1186/1479-5868-3-12

**Published:** 2006-05-30

**Authors:** Kareen Nour, Sophie Laforest, Lise Gauvin, Monique Gignac

**Affiliations:** 1 Ph.D Candidate in Public Health, University of Montreal, Quebec, Canada; Student Member, Groupe de recherche interdisciplinaire en santé (GRIS), University of Montreal; Project Coordinator, CLSC René-Cassin/Institute of Social Gerontology of Quebec, Montreal, Quebec, Canada; 2 Assistant Professor, Department of Kinesiology, University of Montreal; Associate Researcher, Groupe de recherche interdisciplinaire en santé (GRIS), University of Montreal, and CLSC René-Cassin/Institute of Social Gerontology of Quebec, Montreal, Quebec, Canada; 3 Full Professor, Department of Social and Preventive Medicine, University of Montreal; Associate Researcher, Groupe de recherche interdisciplinaire en santé (GRIS), University of Montreal; Researcher, The Léa-Roback Centre on Social Inequalities of Health in Montreal, Montreal, Quebec, Canada; 4 Senior Scientist, Division of Outcomes & Population Health, University Health Network & Associate Professor, Department of Public Health Sciences, University of Toronto, Toronto, Ontario, Canada

## Abstract

**Background:**

This study examined the impact of a home-based self-management intervention for housebound older adults with arthritis on the adoption of health behaviors. The moderating role of socio-demographic, psychological, and physical characteristics in the process of behavior change was also investigated.

**Methods:**

Participants were 113 older adult women (n = 102) and men (n = 11) with osteoarthritis (OA) or rheumatoid arthritis (RA) who were randomly assigned to experimental (n = 68) or wait list control (n = 45) groups. Participants were interviewed using standardized questionnaires at baseline, pre-intervention, and post-intervention.

**Results:**

Adjusted multilevel modeling analyses indicated that from pre to post intervention, experimental participants significantly increased their weekly frequency of exercise and relaxation activities. Socioeconomic status and depression played a moderating role in this change for exercise with larger effects occurring among more privileged, non-depressed participants.

**Conclusion:**

We conclude that a self-management intervention can successfully improve involvement in exercise and relaxation among housebound older adults with arthritis.

## Background

Arthritis is recognized as a major health problem [1-3]. In North America more than 40% of older adults aged 65 and over suffer from arthritis [4,5] Moreover, musculoskeletal problems including arthritis cost the government billions of dollars annually in hospitalizations, treatments, and medications [6,7].

The adoption and maintenance of health behaviors are crucial parts of symptom reduction strategies and therefore, are promoted in arthritis self-management interventions [8]. Regular exercise [9-11], relaxation activities [12], and breathing techniques [13,14] are among the behaviors demonstrated to be effective. Studies show that practice of these behaviors increases the psychological [15,16]. and physical [17,18] well-being of arthritic people. Enjoying leisure activities [19,20] and using everyday coping strategies to maintain energy can also be helpful in reducing symptoms [21] such as pain [22] and fatigue [23-26] Finally, making use of informal and formal social networks [27,28] is related to independence, fewer physical limitations and low levels of depression among older adults with arthritis [29].

One of the first randomized controlled trials evaluating a group-based arthritis self-management intervention [30], *the Arthritis Self-Management Program *(mean age = 60 years), showed that weekly frequency of exercise and relaxation activities among intervention participants significantly increased in comparison to control group participants. The intervention aimed at increasing participants' general knowledge of their disease and symptoms, promoting the adoption/maintenance of pain-reduction strategies (e.g., exercise), and helping participants carry out activities of daily living (e.g., through home adaptations). The intervention's efficacy in changing health behaviors was confirmed by other studies [31,32].

Other randomized controlled trials evaluating "at home" self-management interventions for autonomous people with arthritis have produced similar results regarding behavior change. A six-week videotape and home exercise intervention, *Bone up on your arthritis*, resulted in significant increases in the number of self-care behaviors of arthritic older adults (mean age = 60 years) [33]. The same conclusions were drawn from the randomized controlled trial evaluation of a six-month *Mail-delivered intervention *that consisted of a series of recommendations, videotapes, and documentation on various themes, as it resulted in an increase in exercise frequency for arthritic older adults (mean age = 65 years) [57]. And a randomized evaluation of a *Community-based intervention *where five educational brochures on various topics (nutrition, exercise, etc.) were distributed, showed that the weekly walking frequency of participants (mean age = 56 years) significantly increased after the intervention in comparison with a control group, and that this increase was maintained for 12 months [34].

Although these intervention efforts aimed at decreasing pain, enhancing health, and increasing functioning [3] appear to be helpful in changing behavior among arthritic adults, none of these interventions has been adapted to the needs of an arthritic housebound population that is unable to access community resources. Existing studies have shown that interventions that have demonstrated favorable behavior change, including those conducted at home, targeted younger and more independent older adults (mean ages from 55 to 65 years old) [16-20]. As a result, the generalizability of findings to a more severely afflicted group of arthritic patients is unknown. In fact, one of the major consequences of arthritis is the confinement of older adults to their home. Arthritic older adults who are frail may become housebound [35-37] and frequently suffer from elevated levels of depression and social isolation [38]. Moreover, several prevalence statistics show that the number of older adults confined to their home is increasing slowly [39]. A home-based arthritis self-management intervention called *I'm Taking Charge of My Arthritis! *[40] was designed to address their specific needs, to promote the adoption of health behaviors among this population, and ultimately to improve their psychological [2-16] and physical [32-40] well-being. As with existing self-management interventions, exercise, relaxation, and everyday coping behaviors were targeted. Additional components related to the needs of a more isolated population were added: rediscovering leisure activities and accessing social networks. Indeed, participating in leisure and social activities often dwindles as one becomes more housebound due to the chronicity of the problem (i.e. having pain, difficulties performing activities). By trying to reintegrate social activities into the life of arthritic older adults, the program attempts to break the cycle of isolation. As well, emphasis is placed on the importance of maintaining social contacts by phone, by receiving visits, or by visiting neighbors.

Furthermore, until recently, research evaluating behavior change following an intervention has focused on the frequency of occurrence of behaviors before and after an intervention [31,41] Although this is obviously of critical importance, examining the diversity of activities adopted is also of interest because the process of becoming housebound likely leads to a curtailment of activities. It seems useful to broaden the spectrum of outcome measures to include the variety of activities performed for each category of health behavior.

Also important but studied to a lesser extent, are variables that may moderate or confound behavior change. It seems important to explore whether or not socio-demographic [42-45]., psychological [46-49], and physical [50] characteristics play a moderating role. Several studies have shown that these variables are linked either to the process of coping with arthritis or to the participation rate in health promotion programs, or that they have acted as moderators of change for chronic patients [51-58]

### The current study

Given extant gaps in the literature, the purposes of this study were to: 1) examine the impact of an intervention for housebound older arthritic adults on involvement in exercise, relaxation and leisure activities, everyday coping behaviors, and use and accessibility of social networks, and 2) explore the moderating roles of socio-demographic, physical, and psychological characteristics on changes observed as a result of the intervention. Given previous research, we expected that the intervention would increase the frequency of involvement in health behaviors. Secondly, we anticipated that behavior changes resulting from the intervention would be moderated by participants' socio-demographic and psychological characteristics rather than by their physical characteristics. This expectation is borne from studies on individuals living with chronic pain [59] that show that levels of physical functioning play a minimal role in changes or adoption of new activities whereas one's psychological state such as acceptance and readiness for change plays a major role in moderating behavior change [43,46,60] The current study is part of a larger investigation that showed significant improvements in physical and psychological health as a result of intervention participation [38].

## Methods

### Participants

Participants were recruited over a one-year period through 15 Local Community Health Service Centers located in a large urban center. Case managers identified eligible participants through an examination of their clients' medical records. They contacted potential participants by phone and asked additional eligibility questions. Eligibility criteria were: (a) living at home, (b) being housebound (i.e., not leaving home on one's own more than twice a month), (c) being aged 50 years or older, (d) self-reporting moderate to severe pain during the previous week, (e) suffering from medically diagnosed osteoarthritis (OA) or rheumatoid arthritis (RA), (f) speaking English or French, and (g) experiencing difficulties in performing daily activities. We excluded individuals who: (a) had received a polymyalgia diagnosis; (b) had a recent health problem requiring rehabilitation, or (c) were experiencing cognitive impairment. The initial sample included 125 participants. Between baseline and pre-intervention measurements (eight weeks), 12 participants were lost: two died, four were hospitalized, and six deemed the intervention was too demanding.

### Design and intervention

An experimental study was carried out where participants were randomly assigned to an experimental group (n = 65) or a one-year wait list control group (n = 48). Participants in the experimental group participated in an intervention called *I'm Taking Charge of my Arthritis *[40]. The intervention was based on cognitive-behavioral principles [61,62] and consisted of one-hour weekly home visits by a practitioner for six consecutive weeks. A core of common topics was proposed to all participants. Table 1 presents these topics. Each visit included a review of the previous visit and contract (i.e., setting a weekly goal and developing an action plan), an exploration of a new topic (e.g. pain and stiffness) and the formulation of a new personal contract. Following each visit, practitioners completed a visit report sheet (duration of the visit, themes addressed, and overall problems encountered). Practitioners (n = 10) included occupational therapists and physiotherapists (n = 6), social workers (n = 2) and kinesiologists (n = 2). Although specific calculations of practitioner reliability in intervention implementation were not computed, a variety of procedures was used to ensure consistency. In relation to program participation, the project coordinator ensured that 1) all participants received their six planned meetings, 2) time allotted for these meetings was respected, 3) participants were comfortable with their participation and 4) all questions or comments of participants regarding the program were addressed. Practitioners received a detailed intervention guide and one day of training on intervention implementation provided by the principal investigator. Professional qualifications, assessed according to the current occupation of the practitioner, (e.g.: occupational therapist or physiotherapist) had no effect on participants' satisfaction and perceived efficacy of the intervention at post-program (both rated on Likert scales on the global and specific satisfaction/efficacy of the program from 1 "not satisfied/efficient at all" to 5 "very satisfied/efficient"). Similarly, there were no differences among practitioners in data collected on visit report sheets. In this regard, we noted that few variations existed between intervention design and delivery. Only nine participants felt that they had covered enough materiel before the 6th visit while seven participants received 7 visits because the practitioner needed extra time. Finally, for all participants, the practitioner was the same throughout the intervention and in 93% (n = 54) of cases, topics were addressed in the same order.

**Table 1:** Common Topics of the program *I'm taking charge of my arthritis!*

1	Life with arthritis
2	Exercises and relaxation
3	Better control of stiffness and pain
4	Keep the positive attitude
5	Energy saving strategy
6	Partnership and creativity

### Procedures

The ethics committee of the CLSC René-Cassin which adheres to national ethics guidelines for health research approved and endorsed the study. Participants signed an informed consent form. Eight interviewers who had received a detailed interview guide and systematic training, administered questionnaires in the homes Although specific calculations of inter-interviewer reliability were not computed, a variety of actions and procedures related to the completion of the questionnaire was recorded. In relation to the questionnaire, the project coordinator ensured that 1) all participants were seen at all measurement periods, 2) all items on the questionnaire were completed appropriately, 3) time allotted for interviews was respected, and 4) participants still consented to participate in the study throughout their evaluation. Interviewers obtained an interview guide and participated in a one-day training session. Interview performance was monitored throughout the study by the project coordinator. Interviews were used to measure all variables and lasted two hours. Interviewers were blind to group allocation and to intervention specific objectives. Interviews were conducted upon recruitment (baseline), 8 weeks later prior to randomization (pre-intervention) in order to gauge the stability of measures, and again upon completion of the intervention (post-intervention) using the same questionnaires. In most cases (80%), the same interviewers performed all three interviews with participants. After the pre-intervention measurements, randomization allowed for the allocation of participants into a wait-list control group that received the intervention the second year of the project and an experimental group that received the intervention in the first year.

### Variables and measures

An interview-administered questionnaire was developed to collect information. Although socio-demographic variables were collected only at baseline, all other information was collected in the same manner at three times: baseline, pre-intervention, and post-intervention. Pre-intervention individual's characteristics were used for confounding/moderating analyses.

#### Outcomes variables

The impact of the intervention on five health behaviors was examined, namely exercise, relaxation activities, leisure activities, everyday coping behaviors, and use and accessibility of social networks. In order to assess involvement in these health behaviors, activities falling within each health behavior category were identified. The categorization of activities was based mainly on the content of the intervention and was inspired by the Arthritis Self-Management Behavior Tool [63]. Exercise was categorized into three broad types of activity (i.e., walking, stretching and strengthening) which grouped the diverse physical activities performed by this population. Relaxation included six activities (i.e. distraction, muscle relaxation, breathing exercises, calm music, reading, visualization) and leisure encompassed eight activities (i.e., calling someone, music, cooking, arts and crafts, television, inviting someone to visit, restaurants, reading). Everyday coping behaviors consisted of 13 activities (i.e. establishing priorities, planning the day/week, simplifying and organizing activities, asking for help, maintaining good posture, using assisting devices, trying to sleep better, avoiding a position that causes pain, changing positions, putting pressure on strongest joint, organizing for accessibility, balancing work and rest). The mean weekly frequency over the previous two weeks for exercise, relaxation, and leisure activities was assessed through recall. Participants were asked to estimate the number of times per week they performed each activity (from 0 times/week to 7 times/week). A composite of the total weekly occurrence for each of the three health behaviors was calculated by totaling the weekly frequencies of each activity included in each health behavior category. The maximum score for exercise was 21: the sum of the 3 possible activities for each of the seven days. The maximum score for relaxation activities was 42 and for leisure activities, 56. An index of the variety of activities for each of the three previous health behaviors as well as for everyday coping behavior was computed by dividing the number of different activities recalled by the participant by the total number of activities listed for that behavior. This value was multiplied by 100 to create a percentage. For example, three different exercise activities were defined. If a participant reported only walking, his variety of exercise would be .33 or 33%. Cronbach alphas, calculated for each of the four health behaviors in our sample, were .53 (relaxation), .75 (relaxation and exercises) and .89 (everyday coping behaviors). Finally, the use and accessibility of social networks were evaluated by the Older Americans Resources and Services Scale, consisting of eight questions. Questions pertained to: frequency of seeing friends/family (0 to 7 times/week); frequency of talking to friends/family (0 to 7 times/week); perception that a friend/family would help if needed (yes/no) and for how long (days); presence of a confidant (yes/no); feelings of abandonment by friends/family (yes/no); seeing friends/family as often as desired (yes/no); presence of a person to help or visit (excluding friends/family) (yes/no). One point was allocated to each question for a total ranging from 0 to 8. The sensitivity calculated for our sample was .76, the specificity .71 [64], and the Cronbach alpha 71.

#### Socio-demographic characteristics

Socioeconomic status was estimated using the 1996 Quebec Health and Social Survey question [65,66] Participants identified their perceived socioeconomic status by answering the following question: "How do you perceive your economic situation in comparison to people of your age (from 1 "very high" to 4 "very low")?" Level of education (years recoded to 1 "equal to or more than 9" or 0 "less than 9"), age (years), living situation (1 "alone" or 0 "with someone"), and gender (1 "female" or 0 "male") were also recorded. Participants were asked to indicate if any change had occurred in their living situation since baseline both at pre-intervention and post-intervention.

#### Physical characteristics

The type of arthritis was recorded by the case manager. Considering the number of variables in our analyses as well as the relatively high correlation among the physical characteristics variables, a composite physical factor (from -1 to +1) was created through factorial analysis by combining mean values obtained on pain intensity, fatigue, limitations and stiffness. Pain and fatigue were assessed according to the Visual Analogue Scale, ranging from 0 "none" to 100 "pain/fatigue at their worst possible". Test-retest correlations were .74 for pain and .85 for fatigue [67]. Functional limitations and stiffness were measured by the Western Ontario and McMaster Universities Osteoarthritis Index with a 5-point scale from 0 "none" to 5 "extreme difficulty/stiffness". Functional limitations were evaluated according to 17 items and stiffness according to 2 items. The Cronbach alphas for our sample were .88 and .70 respectively [68]. Higher scores on the factor reflected greater disability. These scales are used by others to evaluate physical health [69-72]

#### Psychological characteristics

For the same reason previously mentioned, a composite psychological factor (from -1 to +1) was built by combining measures of optimism, mastery and self-efficacy. Optimism was measured according to 8 items relating to feelings of control over the situation [73]. Likert-type scale ranged from 1 "strongly disagree" to 5 "strongly agree" The Cronbach alpha calculated for our sample was .81. Mastery was measured by the Pearlin Mastery Scale, a 7 item scale that was included in the Canadian National Health Survey of 1994. It uses the same Likert-type scale as optimism, ranging from 1 to 5. The Cronbach alpha calculated for our sample was .69. For self-efficacy, measures were taken from the Arthritis Self-Efficacy Scale, a 20 item scale ranging from 10 "not certain at all" to 100 "very certain", on participants' confidence about performing various activities. The Cronbach alpha for our sample was .83 [74]. This scale is used in practically every study that examines self-efficacy and arthritis [69,75-77] Self-efficacy is sometimes considered to be a mediator, but in this study it was not the case. Higher scores on the factor reflected greater psychological health [78]. Depression was evaluated separately by the Center for Epidemiological Studies-Depression Scale [79]. Response alternatives ranged from 0 (rarely or none of the time; less than 1 day) to 3 (most or all of the time; 5–7 days). A score of 16 or greater out of 60 was taken as likely presence of depression. The Cronbach alpha for our sample was .80. Participants were dichotomized into depressed (mean scores of 16 and greater on 60) and non-depressed participants (mean scores of less than 17 on 60).

### Statistical analyses

Similar to most community-based intervention studies, the data set included several challenging features [80]. Specifically, although participant dropout from the study was small (10% between baseline and pretest and 13% between pretest and post-test), the initial and final sample sizes differed. In addition, although there were few between group differences, participants in each group were not homogenous at baseline in terms of various parameters. In order to deal with these challenges, we used multilevel modeling techniques through Hierarchical Linear Modeling [81,82] wherein repeated measures (level-1) were conceptualized as nested within persons (level-2). These techniques are viewed as most suitable for repeated measures designs as they allow for the inclusion of data from all participants collected at any time during the investigation (rather than eliminating participants through listwise deletion) and for specifying models that included a random effect at the intercept, thus allowing participants to differ at baseline on outcome measures. Our overall goals in analyzing the data were to determine if the intervention resulted in behavior changes while controlling for potential confounding effects and to explore the presence of moderating influences. Analyses were performed with SPSS (version 10) and HLM 5.04 (Hierarchical Linear Modeling, Scientific Software International, Chicago, IL). Because data were not normally distributed and espoused a Poisson distribution, multilevel modeling procedures for a Poisson distribution were used. Modeling proceeded in three steps. A first set of analyses included parameters operationalizing time (3 measurement times, from T1 to T3 through the inclusion of two level-1 dummy variables) and group membership (one level-2 dummy variable). In this analysis, we were interested in examining whether or not there were changes across time and as a function of group membership. Any changes in the control group between pre-intervention and post-intervention would be reflected in the γ_20 _coefficient. If the change across time between pre-intervention and post-intervention in the experimental group is different from that observed in the control group, then the γ_21 _coefficient will be significant. Second and third sets of analyses were performed by adding possible confounding variables to the models and by testing for moderating influences (not shown in Table 4). Confounding variables included socio-demographic characteristics (continuous measure of age; education with 1 being equal to more than 9 years and 0 being equal to 9 years or less; socioeconomic status with 1 being equal to high perceived socioeconomic status (i.e. high and very high) and 0 being equal to other; psychological characteristics (the psychological factor and depression), and physical characteristics (type of arthritis, with 1 representing OA and 0 representing RA and the physical factor). Our interest was in testing whether or not any intervention effects were maintained despite controlling for possible confounders. Each of the confounders were entered separately in order to clearly examine the impact of these variables on the results and to maintain statistical power. Variables were then entered in blocs and the same results were obtained. For moderating effects, interaction terms between group membership on the one hand, and depression or socioeconomic status on the other hand, were created and entered into the model both with and without control for confound-ding variables. Significant interactions were plotted to allow for interpretation (not shown).

## Results

### Descriptive analyses

After pre-intervention measurements, 113 participants (M = 77.7 years, SD = 10.3) were randomly assigned to experimental (n = 65) or wait list control (n = 48) groups. The 12 participants who dropped out (representing 10% of the initial sample) between baseline and pre-intervention were more likely to have OA (92%) than RA (8.3%) and reported lower exercise involvement than participants maintaining involvement in the study (1 time/week vs. 2.4 times/week) (p = 0.05). Ninety-seven people completed post-intervention measures (58 people in the experimental group 39 wait list control group). This number represents 77.6% of the 125 participants in the initial sample. Those who dropped out between pre-intervention and post-intervention (n = 16) reported fewer everyday coping behaviors (p = 0.05) and higher depression levels (p = 0.04) in comparison to persisters. Most of the pre-intervention participants were women (90%) and 71% lived alone. Almost half of them are reporting less than nine years of education (47%). About sixty-five percent of the sample had been diagnosed with OA and 82% perceived themselves to be financially secure. A greater percentage of participants in the control group reported living in poverty than in the experimental group (26% versus 12%). Participant characteristics at pre-intervention that acted as descriptor, and tested as moderators in our analyze, are presented in table 2. T-tests showed no statistically significant differences between participants in the control and experimental groups at baseline and pre-intervention, except for relaxation practices (p = 0.05).

**Table 2:** Participant Characteristics at Pre-intervention examines as moderator/action

	*Experimental N = 65*		*Control N = 48*		
			
	*M*	*(SD)*	*M*	*(SD)*	*p^(1)^*
Age	77.0	10.4	78.7	10.2	p = .81
Education	9.4	4.1	9.2	4.2	p = .89
Socioeconomic status					
High (%)	87.7		72.9		p = .20
Low (%)	12.3		27.1		p = .12
Composite Psychological factor	-.02	(.93)	-.04	(1.01)	p = .76
Optimism	3.27	(.84)	3.39	(.73)	p = .51
Mastery	2.92	(.61)	2.80	(.73)	p = .44
Self-efficacy	56.67	(14.79)	53.17	(14.38)	p = .61
Depression	19.05	(12.4)	20.21	(11.2)	p = .79
Type of arthritis, OA (%)	58.5		66.7		p = .57
Composite Physical factor	- .03	(-.99)	-.15	(1.02)	p = .16
Pain intensity	65.31	(24.79)	63.65	(22.49)	p = .77
Fatigue	66.00	(28.97)	72.60	(23.83)	p = .40
Functional limitations	3.43	(1.37)	3.31	(1.29)	p = .58
Stiffness	3.11	(.97)	3.24	(.90)	p = .37

^1 ^Comparison of the 2 groups via independent samples t-tests and chi-squares for categorical variables.

### Impact of the intervention

Table 3 presents observed values and relative increases for all outcome variables across experimental and control groups at different times. Results of multilevel modeling analyses are described below and depicted in table 4.

**Table 3:** Observed Values on Outcome Variables at Different Times.

Measure time	Baseline			Pre-intervention		Post-intervention		Relative increase ^1^	
			
Outcome variables	Max	*Exp*.	*Control*	*Exp*.	*Control*	*Exp*.	*Control*	*Exp*.	*Control*
Group									
				
Occurrence of exercise	21	7.35	7.12	6.48	4.79	10.02	5.64	54.5	17.5
• Occurrence of stretching exercises	7	2.85	2.73	2.56	2.06	4.57	2.30	78.5	12.1
• Occurrence of walking	7	2.26	2.83	2.04	1.77	2.57	1.72	25.4	-2.8
• Occurrence of strengthening exercises	7	2.25	1.56	1.87	.96	2.88	1.61	54.0	67.7
Variety of exercises	1	.47	.49	.47	.38	.63	.41	34.0	7.9
Occurrence of relaxation activities	42	3.66	6.81	5.92	6.23	6.66	6.02	12.4	-3.2
Variety of relaxation activities	1	.11	.20	.17	.17	.18	.19	5.9	11.8
Occurrence of leisure activities	56	12.22	10.85	9.43	13.02	10.12	12.13	7.32	- 6.84
Variety of leisure activities	1	.25	.25	.24	.30	.25	.29	4.21	-3.30
Variety of everyday coping behaviors	1	.64	.67	.69	.69	.74	.74	7.20	7.20
Social networks	8	4.81	4.86	4.66	4.49	4.32	4.38	-7.32	-2.45

^1 ^Predicted relative increase computed by subtracting post-intervention values from pre-intervention values and dividing this value by the pre-intervention value.

**Table 4:** Multilevel Modeling Analyses Predicting Health Behavior after an Intervention and the Cross-Level Interaction between Individual Characteristics and Times

		Baseline			Pre-intervention Change from Baseline			Post-intervention Change from Baseline		
			
Within-Subject Fixed Effects		*Parameter*	*Coeff*.	*SE*	*Parameter*	*Coeff*.	*SE*	*Parameter*	*Coeff*.	*SE*
			
Occurrence of exercise	Intercept	γ_00_	1.66	.15	γ_10_	-.39	.09	γ_20_	-.30	.09
	Group	γ_01_	.07	.20	γ_11_	.25	.11	γ_21_	.58*	.11
Variety of exercises	Intercept	γ_00_	.40	.13	γ_10_	-.25	.19	γ_20_	-.14	.19
	Group	γ_01_	-.07	.17	γ_11_	.22	.24	γ_21_	.43*	.24
- Occurrence of stretching	Intercept	γ_00_	.56	.19	γ_10_	-.27	.14	γ_20_	-.18	.14
	Group	γ_01_	.19	.24	γ_11_	.11	.18	γ_21_	.67*	.17
- Occurrence of walking	Intercept	γ_00_	.56	.22	γ_10_	-.49	.14	γ_20_	-.72	.16
	Group	γ_01_	-.22	.29	γ_11_	.41	.19	γ_21_	.76*	.21
- Occurrence of strengthening exercises	Intercept	γ_00_	-.48	.29	γ_10_	-.42	.19	γ_20_	.05	.18
	Group	γ_01_	.60	.37	γ_11_	.20	.24	γ_21_	.14	.22
- Occurrence of relaxation activities	Intercept	γ_00_	1.17	.26	γ_10_	-.15	.09	γ_20_	-.20	.09
	Group	γ_01_	-.89	.35	γ_11_	.54	.13	γ_21_	.79*	.13
Variety of relaxation activities	Intercept	γ_00_	.07	.18	γ_10_	-.17	.19	γ_20_	-.09	.20
	Group	γ_01_	-.69	.26	γ_11_	.60	.29	γ_21_	.60	.22
Occurrence of leisure activities	Intercept	γ_00_	1.67	.12	γ_10_	.35	.08	γ_20_	.23	.09
	Group	γ_01_	.09	.15	γ_11_	-.34	.11	γ_21_	-.29	.12
Variety of leisure activities	Intercept	γ_00_	.17	.14	γ_10_	.37	.18	γ_20_	.29	.19
	Group	γ_01_	.05	.18	γ_11_	-.27	.24	γ_21_	-.07*	.25
Variety of everyday coping behaviors	Intercept	γ_00_	2.14	.06	γ_10_	-.00	.07	γ_20_	.09	.07
	Group	γ_01_	-.02	.07	γ_11_	.05	.09	γ_21_	.02	.09
Social networks	Intercept	γ_00_	6.48	.28	γ_10_	-.46	.18	γ_20_	-.09	.19
	Group	γ_01_	-.16	.37	γ_11_	.25	.24	γ_21_	.29	.26

* p < .05

Significance of coefficient γ_21 _is indicative of intervention effects using the following model:

Level 1 model: Outcome = β_0j _+ β_1j_X_1 _+ β_2j_X_2_+ r_ij_

Level 2 model: T_1 _β_0j _= γ_00 _+ γ_01 _Gr_j _+ u_oj_

T_2 _β_1j _= γ_10 _+ γ_11 _Gr_j_

T_3 _β_2j _= γ_20 _+ γ_21 _Gr_j_

Meaning of Coefficients:

γ_00 _= Predicted value in outcome variable at baseline for participants in control group

γ_01 _Gr_j _= Predicted difference in outcome variable at baseline for participants in experimental group

γ_10 _= Predicted change in outcome variable from baseline to pre-intervention for participants in control group

γ_11 _Gr_j _= Predicted difference in change in outcome variable from baseline to pre-intervention for participants in experimental group

γ_20 _= Predicted change in outcome variable from baseline to post-intervention for participants in control

γ_21 _Gr_j _= Predicted difference in change in outcome variable from baseline to post-intervention for participants in experimental group.

where i: 1 .....N individuals ; r_ij_: level 1 error term ; γ : parameter coefficients ; u_oj_: level-2 random effect ; β: coefficient; Gr: group membership (1 = experimental, 0 = control) ; X_1_: dummy variable (1 = pre-intervention, 0 = otherwise) ; X_2_: dummy variable(1 = post-intervention, 0 = otherwise)

For weekly occurrence of exercise, observed means indicated that control participants changed their weekly occurrence of exercise from 4.79 (SD = 5.11) (max = 21) times per week at pre-intervention to 5.64 (SD = 5.81) times at post-intervention, whereas experimental participants increased from 6.48 (SD = 6.37) times per week to 10.02 (SD = 6.65). Multilevel modeling analyses confirmed that these differential change patterns were statistically significant (p < .001) and that the addition of confounding variables did not attenuate this effect. In addition, moderating influences were observed. Results showed that although the intervention increased the weekly occurrence of exercises for among all experimental participants (p < .001), participants with a higher socioeconomic status showed greater improvements (p < .001). Furthermore, depression significantly moderated the impact of intervention. In fact, experimental participants without depression showed improvements in weekly occurrence of exercise whereas experimental participants with depression showed no improvements (p < .001).

For variety of exercises, the intervention did not have a significant influence although there was a trend in this direction (p = .07) with experimental participants group non significantly increasing the variety of exercises by 34% between pre-intervention and post-intervention in comparison to 8% for control group participants. No confounding or moderating effects were observed.

Further analyses were conducted on the three activities of the exercise measure namely, walking, stretching, and strengthening. From pre-intervention to post-intervention, experimental participants changed their weekly stretching frequency from 2.56 (SD = 2.92) to 4.57 (SD = 2.94) times a week whereas control participants changed it from 2.06 (SD = 2.80) to 2.30 (SD = 3.00) times a week. Multilevel modeling analyses showed a significantly larger increase in stretching frequency for experimental participants (p < .001). The addition of confounding variables to the model did not attenuate this effect. None of the moderating influences were statistically significant. For walking frequency, differences were not statistically significant between groups (p = .08). However, between pre-intervention and post-intervention a 3% decrease for control participants and a 25% increase among experimental participants were observed. There were no statistically significant confounding or moderation effects. Finally, for strengthening frequency, the intervention had no significant impact (p = .52). No confounding or moderating effects were observed. For weekly occurrence of relaxation activities, control group participants decreased from 6.23 times per week (SD = 6.86) (max. of 42) at pre-intervention to 6.02 (SD = 6.63) at post-intervention, whereas experimental group participants changed from 5.92 (SD = 7.23) times per week at pre-intervention to 6.66 (SD = 8.03) at post-intervention. Multilevel modeling analyses showed that experimental participants had a significantly larger increase in their weekly occurrence of relaxation activities (p = .05.). Control for confounding variables did not change these effects and no moderating effects were observed.

For variety of relaxation exercises, observed means did not change from pre-intervention to post-intervention for either control (from 17% to 19%) or experimental participants (from 17% to18%) (p = .40). No confounding or moderating effects were observed.

For weekly occurrence of leisure activities, observation of means indicated that participants in the control group decreased involvement from 13.02 (SD = 8.05) times per week at pre-intervention to 12.13 (SD = 7.83) at post-intervention, whereas experimental group participants increased from 9.43 (SD = 7.77) at pre-intervention to 10.12 (SD = 8.20) at post-intervention. Multilevel modeling analyses showed that although patterns of change from pre-intervention to post-intervention were different across experimental and control groups (p = .02), the actual frequency of leisure activities at post-intervention was not different across experimental and control group participants (p = .51) as control group participants reported significantly greater frequency of leisure activities at pre-intervention. No confounding or moderating effects were found

For variety of leisure activities, the intervention did not result in significant changes although there was a tendency (p = .07) for experimental participants to show an increase and for control participants to show a decrease. No confounding nor moderating effects were observed.

The intervention did not result in significant improvements in the variety of everyday coping behaviors (p = .83). Similarly, no significant effects were observed for the predicted use and accessibility of social networks (p = .25) both with and without controlling for confounding variables. Analyses showed no moderating influences for either of the two variables.

## Discussion

Given the dearth of information on the impact of health promotion interventions on the health behavior of more severely ill arthritic patients, the purposes of this study were to examine the impact of an intervention designed to address the needs of housebound older arthritic adults on exercise, relaxation and leisure activities, everyday coping behaviors, and use and accessibility of social networks and to explore the moderating roles of socio-demographic, physical, and psychological characteristics on changes observed as a result of the intervention. Results regarding health behaviors are clear and convincing. First, multilevel modeling analyses showed that the intervention significantly increased weekly occurrence of exercises (particularly stretching exercises and walking). Group differences remained significant after adjustment for confounding variables. Moreover, moderating effects of depression and socioeconomic status were observed with non-depressed participants accruing benefits, depressed participants not improving, and smaller improvements among participants with lower perceived socioeconomic status. A trend was also observed for an increase in the diversity of exercises practiced. These findings suggest that severely ill patients can benefit from health promotion efforts such as the program *I'm taking charge of my arthritis! *in order to change their health behaviors.

These findings parallel those reported in previous studies evaluating arthritis self-management interventions with autonomous arthritic participants [31,83-85] As in those studies, the frequency of exercises increased after a self-management program [86]. However, our results go one step further considering that we observed significant changes not only in the global frequency of practice of exercises, but also in the frequency of particular exercise activities and in the variety of exercises performed (types of variables not studied before). Given the experimental design, we attribute these changes to the intervention content [87]. In fact, the intervention included stretching and strengthening activities as well as encouragement to walk in the home and immediate surroundings [88]. Such reinforcement might have produced these significant results. Anecdotally, participants reported that the exercise session was their favorite. Others have reported similar findings [89].

However, unexpected results were observed for strengthening exercises where both groups increased weekly involvement. The reasons underlying this finding remain obscure and require further investigation. Furthermore, a significant change following the intervention was observed for frequency of relaxation activities (i.e. muscle relaxation, breathing exercises, music, reading and visualization). However, the meaning of this result is more ambiguous due to pre-intervention differences. On the one hand, the intervention appeared to increase the weekly occurrence of relaxation activities for experimental participants which provided justification for the allocation of a session for this topic. On the other hand, despite randomization of participants to groups, persons in the experimental group reported significantly fewer relaxation activities at baseline and pre-intervention than the participants in the wait-list control group. At post-intervention, the two groups were not significantly different from one another on this variable. These results could be due to bone fide intervention effects or to "regression toward the mean" [95]. Further research into natural trajectories of involvement in exercise as a function of states of disability could shed light on this question.

It is also interesting that, following the intervention, the frequency of exercise and relaxation activities increased but their variety did not. In fact, it appears more difficult to introduce a new exercise than to increase the frequency of an activity that is already part of the person's repertoire [90]. The implication is that future interventions might focus on extant activities rather than on attempts to introduce new ones even though introducing new activities might be beneficial if some have to be given up or limited.

The intervention did not significantly change everyday coping behaviors, participation in leisure activities, and the use and accessibility of social networks. Other studies have not focused on these behaviors which renders comparisons difficult. However, a number of factors might account for these findings. First, there may be a ceiling effect for everyday coping behaviors. In fact, at baseline, many of the participants reported engaging in a large number of everyday coping behaviors which is not surprising considering that participants had access to home care practitioners prior to and during the intervention whose continued care targeted coping behaviors. Also, participating in the study seemed to encourage both groups to use their coping behaviors even more. A ceiling effect may also be responsible for the lack of change in the use and accessibility of social networks even though 70% of participants lived alone. Presence of home-care health professionals and volunteers may explain these effects. Furthermore, the extra attention they received from case managers, interviewers, and the project coordinator throughout the study may explain participants' high scores.

With respect to leisure activities (e.g. reading, arts and craft, inviting someone to visit, etc.), lack of improvement might be partially explained by the fact that a small portion of the intervention is devoted to this topic and that some leisure activities require greater effort or another person (e.g. playing cards). Given that leisure has a positive impact on the life of arthritic older adults since it breaks the social isolation and distracts them from focusing on their pain [20], time and effort should be devoted in future interventions to improving participation in leisure activities [91].

As anticipated, selected behavior changes (e.g., weekly occurrence of exercise) were moderated by socioeconomic status and depression. This finding has important implications for setting intervention priorities and timing. For example, the results suggest that greater attention and/or treatment efforts should be paid to depressive symptoms before initiating other efforts to manage arthritis [92]. Because financial concerns make life even more stressful, activities other than those related to basic needs are not likely to be a priority for low-income participants [93]. Parallel resources or allocation of additional funds to the intervention could help decrease stress and allow time and freedom for other activities [43]. Also of interest was that none of the physical characteristics, when considered as a composite (pain, stiffness, limitations and fatigue), was found to moderate behavior change which suggests that older adults with varying degrees of physical limitation benefited equally from the intervention. This challenges the notion that older frail adults are unlikely to change [90].

There are a number of strengths and limitations to this study. Its strengths include the randomized controlled trial design, the novel statistical analyses used, and the inclusion of baseline data prior to pre-intervention data. One limitation has do to with the sample size: a sample of over one hundred participants is large considered for the small target population but remains small for statistical analyses purposes. In addition, the self-report rather than observational measures of health behaviors are likely to include measurement error even though it is widely used [94]. Moreover, we must recognize that there might be a measurement problem regarding the number of activities for a certain behavior which can play a role in finding significant intervention effects. In future studies, these numbers should be equal for each behavior. We also note that this study was conducted in Canada where all residents have access to subsidized health care. It is unclear whether or not findings can be generalized to other health care systems. Finally, findings on moderators should be replicated in order to better understand the reasons underlying the effects on some behaviors and not on others.

In terms of practical implications, we think that this "ready-to-use" intervention could be useful for health professional working in homecare services or other agencies (e.g., rehabilitation, private or public agencies, support groups); as is done by the Ontario Arthritis Society which offers all interventions related to arthritis. In this regard, we emphasize that complete access to subsidized health care does not address all the health needs of housebound arthritic older adults. Rather health promotion interventions such as *"I'm taking charge of my arthritis!" *offer an important adjuvant to health care. In this regard, Lorig et al. (2001) showed that self-management interventions reduce (in a ratio of 1:4) the costs of each arthritic person to the health care system due to reductions in the number of visits to physicians and health care professionals as well as reductions in the number of days of hospitalization. Further research should explore the implementation of the intervention by different agencies in order to evaluate if behavior change is maintained regardless of the context of application. As well, the content and structure of a self-management intervention that might influence behavior change should be examined [87].

## Conclusion

Self-management interventions can successfully increase exercise and relaxation activities among housebound older adults with arthritis. However, practitioners and program developers should keep in mind that psychological state and economic situation play important roles in how people become involved in these programs and the degree which they change their habits.

## Competing interests

The author(s) declare that they have no competing interests.

## Authors' contributions

KN and SL jointly conceptualized the research project, participated in the design of the study and wrote the article. KN further developed the ideas and the statistical analysis and coordinated the gathering of data. SL, as principal researcher of the project *I'm Taking Charge of My Arthritis!*, undertook the general supervision of the project and actively participated in each step. LG primarily guided the statistical analysis and contributed to the write-up of the manuscript. MG made a significant contribution to writing of the article, through a critical review of the intellectual content. All authors read and approved the final manuscript.
